# Molten salt synthesis of disordered spinel CoFe_2_O_4_ with improved electrochemical performance for sodium-ion batteries

**DOI:** 10.1039/d3ra07050f

**Published:** 2023-11-22

**Authors:** Sarah Umeera Muhamad, Nurul Hayati Idris, Hanis Mohd Yusoff, Muhamad Faiz Md Din, Siti Rohana Majid, Lukman Noerochim

**Affiliations:** a Energy Storage Research Group, Faculty of Ocean Engineering Technology and Informatics, Universiti Malaysia Terengganu 21030 Kuala Nerus Terengganu Malaysia nurulhayati@umt.edu.my; b Faculty of Science and Marine Environment, Universiti Malaysia Terengganu 21030 Kuala Nerus Terengganu Malaysia; c Advance Nano Material (ANOMA) Research Group, Faculty of Science and Marine Environment, Universiti Malaysia Terengganu 21030 Kuala Nerus Terengganu Malaysia; d Department of Electrical & Electronic Engineering, Faculty of Engineering, National Defence University of Malaysia Kem Sungai Besi 57000 Kuala Lumpur Malaysia; e Centre for Ionics University of Malaya, Department of Physics, Faculty of Science, University of Malaya 50603 Kuala Lumpur Malaysia; f Department of Materials and Metallurgical Engineering, Institut Teknologi Sepuluh Nopember Surabaya 60111 Indonesia lukman@mat-eng.its.ac.id

## Abstract

Sodium-ion (Na-ion) batteries are currently being investigated as an attractive substitute for lithium-ion (Li-ion) batteries in large energy storage systems because of the more abundant and less expensive supply of Na than Li. However, the reversible capacity of Na-ions is limited because Na possesses a large ionic radius and has a higher standard electrode potential than that of Li, making it challenging to obtain electrode materials that are capable of storing large quantities of Na-ions. This study investigates the potential of CoFe_2_O_4_ synthesised *via* the molten salt method as an anode for Na-ion batteries. The obtained phase structure, morphology and charge and discharge properties of CoFe_2_O_4_ are thoroughly assessed. The synthesised CoFe_2_O_4_ has an octahedron morphology, with a particle size in the range of 1.1–3.6 μm and a crystallite size of ∼26 nm. Moreover, the CoFe_2_O_4_ (M800) electrodes can deliver a high discharge capacity of 839 mA h g^−1^ in the first cycle at a current density of 0.1 A g^−1^, reasonable cyclability of 98 mA h g^−1^ after 100 cycles and coulombic efficiency of ∼99%. The improved electrochemical performances of CoFe_2_O_4_ can be due to Na-ion-pathway shortening, wherein the homogeneity and small size of CoFe_2_O_4_ particles may enhance the Na-ion transportation. Therefore, this simple synthetic approach using molten salt favours the Na-ion diffusion and electron transport to a great extent and maximises the utilisation of CoFe_2_O_4_ as a potential anode material for Na-ion batteries.

## Abstract 2

Octahedron morphology of CoFe_2_O_4_ is obtained with particle size in the range of 1.0–1.5 μm. CoFe_2_O_4_ exhibits a high discharge capacity of 839 mA h g^−1^ in the first cycle at a current density of 0.1 A g^−1^ and good rate capability.

## Graphical abstract

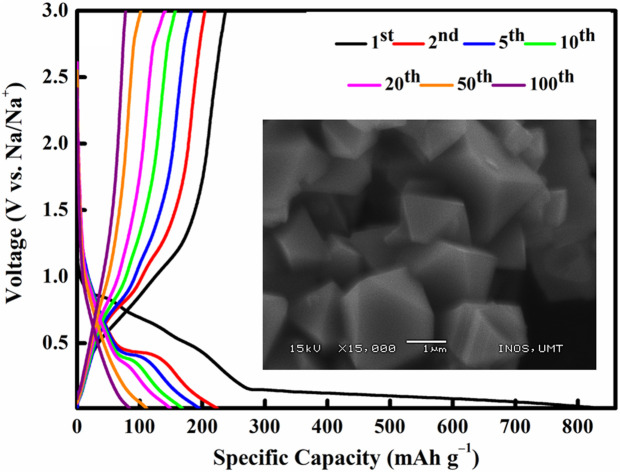

## 1. Introduction

Recently, sodium-ion (Na-ion) batteries have gained popularity owing to the abundance and widespread distribution of sodium resources, low cost and similarity in terms of electrochemistry with lithium-ion (Li-ion) batteries.^1–5^ However, finding ideal anode materials for Na-ion batteries remains challenging because Na-ions have a greater ionic radius of 1.02 Å compared to 0.76 Å of Li-ions, affecting phase stability and decreasing ion/electron transport properties.^6–8^ Additionally, low energy density is another drawback of Na-ions, because sodium is heavier (23 g mol^−1^) and has a lower redox potential (−2.71 V *vs.* standard hydrogen electrode (SHE)) compared to lithium (−3.02 V *vs.* SHE).^9^ To address these issues, determining a suitable anode material is essential. Designing anodes with materials having a high specific capacity, high conductivity and high sodium storage capacity has proven to be a successful strategy for Na-ion batteries.^10,11^ Previously, various materials,^12–14^ including transition metal oxides (TMOs) such as Mn_2_O_3_,^15^ Mn_3_O_4_,^16^ Fe_3_O_4_^17^ and Co_3_O_4_^18^ have been investigated for synthesising anodes for Na-ion batteries. However, TMOs possess low electrical conductivity and exhibit large volume change because the active material particles swell and shrink in response to the insertion and extraction of Na-ions during charging and discharging.^19^

Recently, various studies have focused on iron-based (Fe-based) oxide anode materials^20–22^ and spinel ferrites, with the formula AFe_2_O_4_ (A = Mn, Co, Cu and Ni), are considered to display greater performance than simple iron oxide and to have the advantages of natural abundance, non-toxicity and cost efficiency.^23,24^ Importantly, spinel ferrite demonstrated a remarkable synergetic effect and high capacity.^25–27^ Based on a previous report,^10^ MgFe_2_O_4_ was synthesised using a microwave-assisted method, demonstrating outstanding electrochemical performance and good cyclability. In MgFe_2_O_4_, spinel ferrite acts as a buffer for the matrix to maintain structural stability and reduce the effect of volume change during charging and discharging.^28,29^ Additionally, the spinel ferrite performs better than single oxide and has higher electrical conductivity.^30,31^

Interestingly, cobalt ferrite (CoFe_2_O_4_) has attracted research attention as a potential anode material for Na-ion batteries. CoFe_2_O_4_ consists of two metal ions capable of accepting multiple electrons, demonstrating superior performance with a high theoretical capacity of 916 mA h g^−1^.^32^ Zhang *et al.*^33^ reported the porous CoFe_2_O_4_ nanocubes delivered a high capacity of 360 mA h g^−1^ after 50 cycles and displayed a high initial coulombic efficiency of 68.8%. In another work, He *et al.*^23^ synthesised CoFe_2_O_4_ through a hydrothermal technique. In the first cycle, the discharge capacity of the CoFe_2_O_4_ was 300 mA h g^−1^ (current density of 100 mA g^−1^); however, the capacity faded rapidly. Similarly, Feng *et al.*^34^ synthesised CoFe_2_O_4_*via* hydrothermal method and demonstrated a discharge capacity of approximately 200 mA h g^−1^ (at a current density of 0.05 A g^−1^) after 90 cycles. Hence, further research needs to be conducted to enhance the electrochemical performance of CoFe_2_O_4_-based anodes, which can be accomplished by exploring synthesis different synthesis methods because synthesis methods can impact electrochemical performances.

To date, different methods have been developed for synthesising CoFe_2_O_4_, including hydrothermal,^35^ mechanical-alloying^36^ and ball-milling^37^ methods. The synthesis methods can affect the structure, properties, morphologies, phase purity and crystallinity of CoFe_2_O_4_.^38^ The molten salt method may offer more advantages compared to other methods, such as well-defined facets despite reactions taking place at lower temperatures within a short time, highly homogeneous product formation and reduced particle agglomeration.^39,40^ Besides, numerous studies have been reported on the synthesis of CoFe_2_O_4_*via* the molten salt method with various salt combinations. Yang *et al.*^41^ used Li_2_SO_4_/Na_2_SO_4_ and NaCl/KCl to synthesise CoFe_2_O_4_ using the molten salt method for the first time. The CoFe_2_O_4_ particles are well formed and many particles have octahedron shape, indicating that an interface reaction mechanism regulates particle growth. Another study demonstrated that CoFe_2_O_4_ synthesised through the molten salt method using NaCl and KCl exhibited excellent electrochemical performance in Li-ion batteries with good cyclability and high reversible capacity.^42^

Herein, we report that the CoFe_2_O_4_ synthesised *via* the molten salt method using NaCl and KCl as precursors yields a remarkable electrochemical performance as an anode material in Na-ion batteries. During the synthesis, the molten salt helps control the particle size and shape at low temperatures and protects particles from agglomeration, resulting in homogeneous particles. The most important aspect of this structure is the octahedron shape of the CoFe_2_O_4_ particle, which is between 1.1 and 3.6 μm in size and can provide sites for reaction with Na-ions.^43^ The unique structure of the octahedron CoFe_2_O_4_ particle notably enhanced the electrochemical performance of CoFe_2_O_4_, with a high initial discharge capacity of 839 mA h g^−1^ and capacity retention of 98 mA h g^−1^ at 0.1 A g^−1^ after 100 cycles, indicating the remarkable potential of CoFe_2_O_4_ as an anode material.

## 2. Experimental

### 2.1 Synthesis of spinel CoFe_2_O_4_

A spinel CoFe_2_O_4_ was synthesised by adapting a previously described molten salt method.^44^ Highly pure cobalt(ii) chloride hexahydrate (CoCl_2_·6H_2_O, ≥99.0%), ferric chloride (FeCl_3_, ≥98%), potassium chloride (KCl, 99.5%) and sodium chloride (NaCl, 99.5%) purchased from Sigma-Aldrich were ground with hydrogen peroxide (H_2_O_2_, 30%) purchased from Merck Millipore using an agate mortar, and heated overnight at 120 °C under vacuum. The CoFe_2_O_4_ powders were obtained by calcinating them at 700 °C, 800 °C and 900 °C for 6 h at a heating rate 5 °C min^−1^ in air and then labelled as M700, M800 and M900, respectively. Finally, the powders were washed, filtered and dried at 100 °C for 12 h (under vacuum).

### 2.2 Material characterisation

X-ray diffraction (XRD) pattern of the sample was obtained using Rigaku Miniflex II under monochromatic Cu-Kα radiation (*λ* = 1.5148 Å) from 5° to 80°. The morphology and structure of the CoFe_2_O_4_ were viewed using scanning electron microscopy (SEM; JEOL JSM-6360LA) and transmission electron microscope (TEM; TECNAI G2 F20). The Fourier-transform infrared (FTIR) spectra were obtained using Shimadzu IR Tracer-100. The X-ray photoelectron spectroscopy (XPS) was used to examine the chemical states of the elements present in the CoFe_2_O_4_ using an Axis Ultra DLD XPS, Kratos and obtained spectra were fitted using CASA software. Then, Raman spectroscopy (Renishaw) was performed using 532 nm excitation extended with 0.1% power-laser measurements.

### 2.3 Electrochemical measurements

All chemicals were obtained from Sigma-Aldrich. The CoFe_2_O_4_ electrode is prepared by mixing 75 wt% active material, 15 wt% carbon black and 10 wt% polyvinylidene fluoride (PVDF) in *N*-methyl-2-pyrrolidone (NMP). The slurry was applied onto a copper foil with an electrode mass loading of ∼2 mg cm^−2^ and dried at 100 °C overnight. The coin-type cell (CR2032) was assembled in an argon-filled glove box (MBRAUN Unilab) with sodium metal as the counter electrode and glass fibre as the separator. The electrolyte solution was prepared using 1 M NaClO_4_ (98%) in a mixture of propylene carbonate (anhydrous, 99.7%) with 5 wt% fluoroethylene carbonates (99%). Cyclic voltammetry (CV; CHI 700E) and galvanostatic charge and discharge (NEWARE battery analyser) were controlled in a range of 0.01–3.00 V voltage *versus* Na/Na^+^.

## 3. Results and discussion

**Fig. 1:**
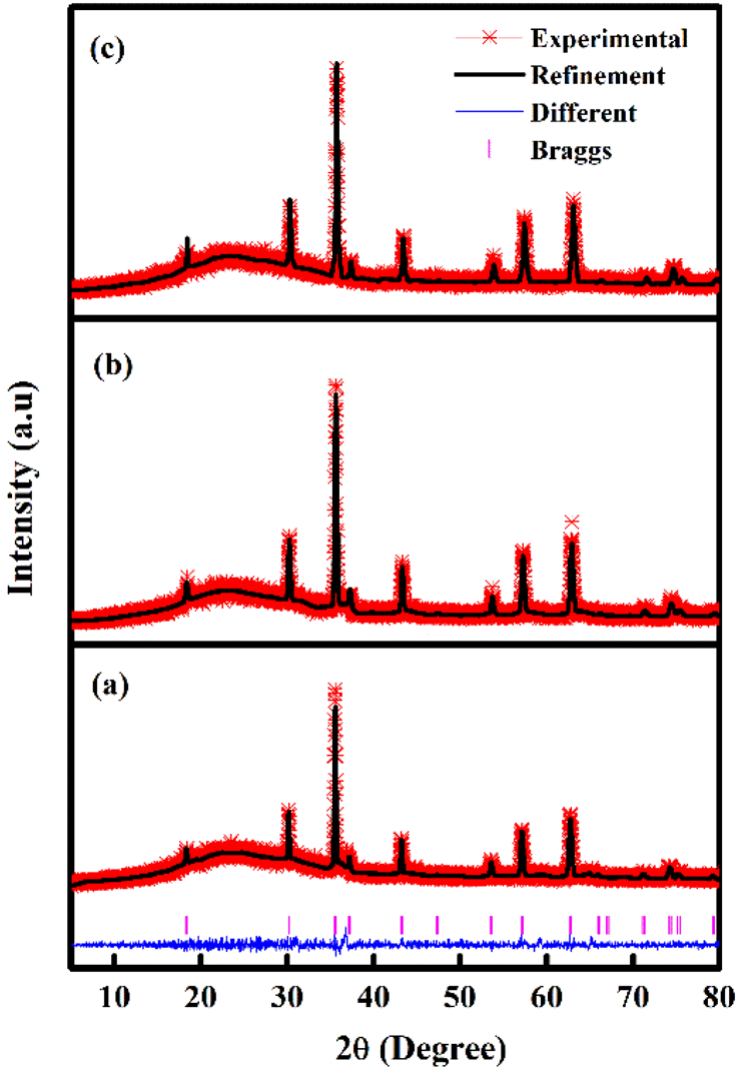
Rietveld refinements fits of the XRD data of the CoFe_2_O_4_ samples (a) M700 (b) M800 and (c) M900.

Table 1 and Fig. 1 display all the XRD patterns and Rietveld refinement profiles of all the samples. All diffraction peaks can be readily indexed as cubic spinel of CoFe_2_O_4_, which agrees well with the conventional CoFe_2_O_4_ spinel with the *Fd*3*m* space group (JCPDS no. 22-1086).^45^ No additional peaks are observed, demonstrating the purity of the CoFe_2_O_4_ produced. Overall, the intensity peaks become sharper as the calcination temperature increases, indicating that the crystallite size increases with temperature.^46^ The crystallite sizes (*L*) for all samples were calculated using Scherrer's equation:1
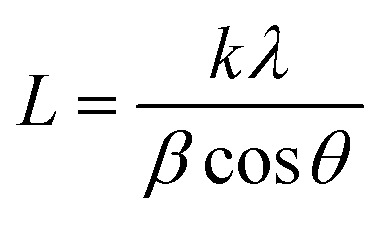
where, *k* is a constant (0.9394), *λ* is the Cu-Kα radiation wavelength (1.5148 Å), *β* is the full width at half-maximum on the XRD peak in radians and *θ* is the angle of diffraction. M700, M800 and M900 crystallite sizes were calculated to be 26.26, 29.84 and 31.36 nm, respectively. Moreover, the lattice parameters, *a* for the calcination treatment for M700, M800 and M900 slightly decrease by 8.37, 8.36 and 8.33 Å, respectively. This phenomenon is typically occurs due to the defects removal such as oxygen vacancies and the lattice contracts during calcination.^47^ These findings are similar to the results from a previous study.^48^

**Table 1:** Rietveld refinements results for M700, M800 and M900

Sample	*a* (Å)	*c* (Å)	Bragg *R*_factor_ (%)	*R*_f_ factor (%)	*χ* ^2^
M700	8.3701(116)	8.3701(116)	10.6	10.6	0.30
M800	8.3567(109)	8.3567(109)	18.5	11.9	0.91
M900	8.3317(90)	8.3317(90)	19.4	12.5	0.97

**Fig. 2:**
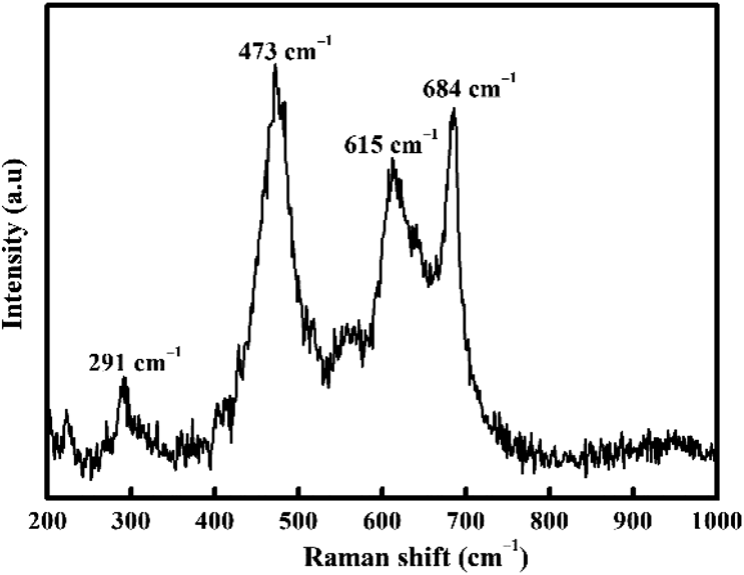
Raman spectroscopy of CoFe_2_O_4_ for M800.

Raman spectroscopy (Fig. 2) was also carried out to confirm the nature of CoFe_2_O_4_. Inverse spinel CoFe_2_O_4_ shows an A_1g_ symmetry at 684 cm^−1^ associated with the tetrahedral sub-lattice and octahedral sub-lattice at the peak at 615 cm^−1^.^49,50^ The band at 473 cm^−1^ is attributed to asymmetric bending of Fe (Co)–O.^50^ Conversely, the Raman band at 291 cm^−1^ is attributed to the E_g_ symmetric bending of Fe (Co)–O.^51^

**Fig. 3:**
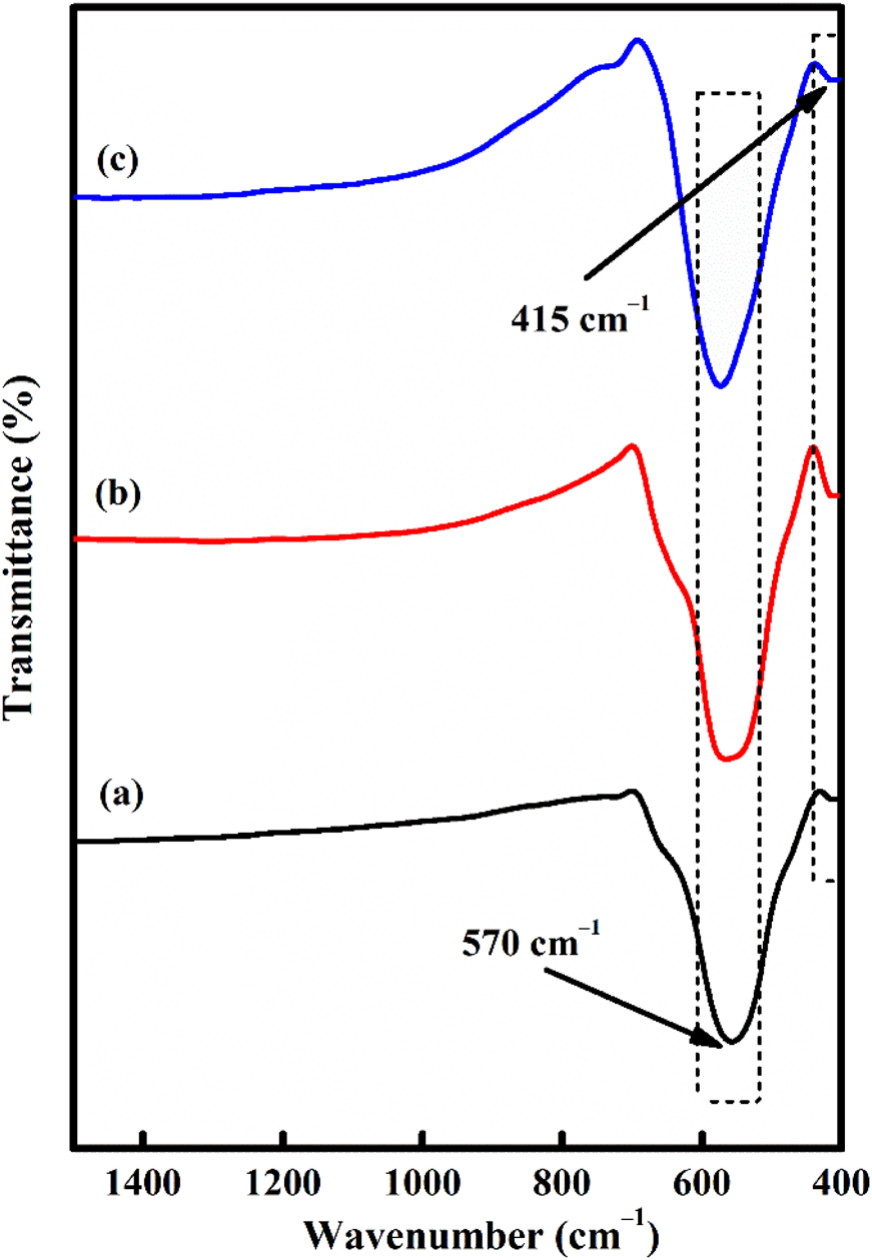
FTIR spectra of CoFe_2_O_4_ for (a) M700, (b) M800 and (c) M900.

The formation of CoFe_2_O_4_ spinel was also supported by the FTIR spectra (Fig. 3). The appearance of two peaks at 570 and 415 cm^−1^ is closely linked to the stretching vibrations of metal oxide in the octahedral site Co^2+^–O^2−^ and tetrahedral site Fe^3+^–O^2−^, respectively.^52,53^ These two typical bands can be detected in almost all CoFe_2_O_4_ structures.^54^ However, at relatively higher temperatures, the peaks become sharper and narrower due to lattice distortion minimization and improve the crystallinity.^55^ This fact is in agreement with XRD.

**Fig. 4:**
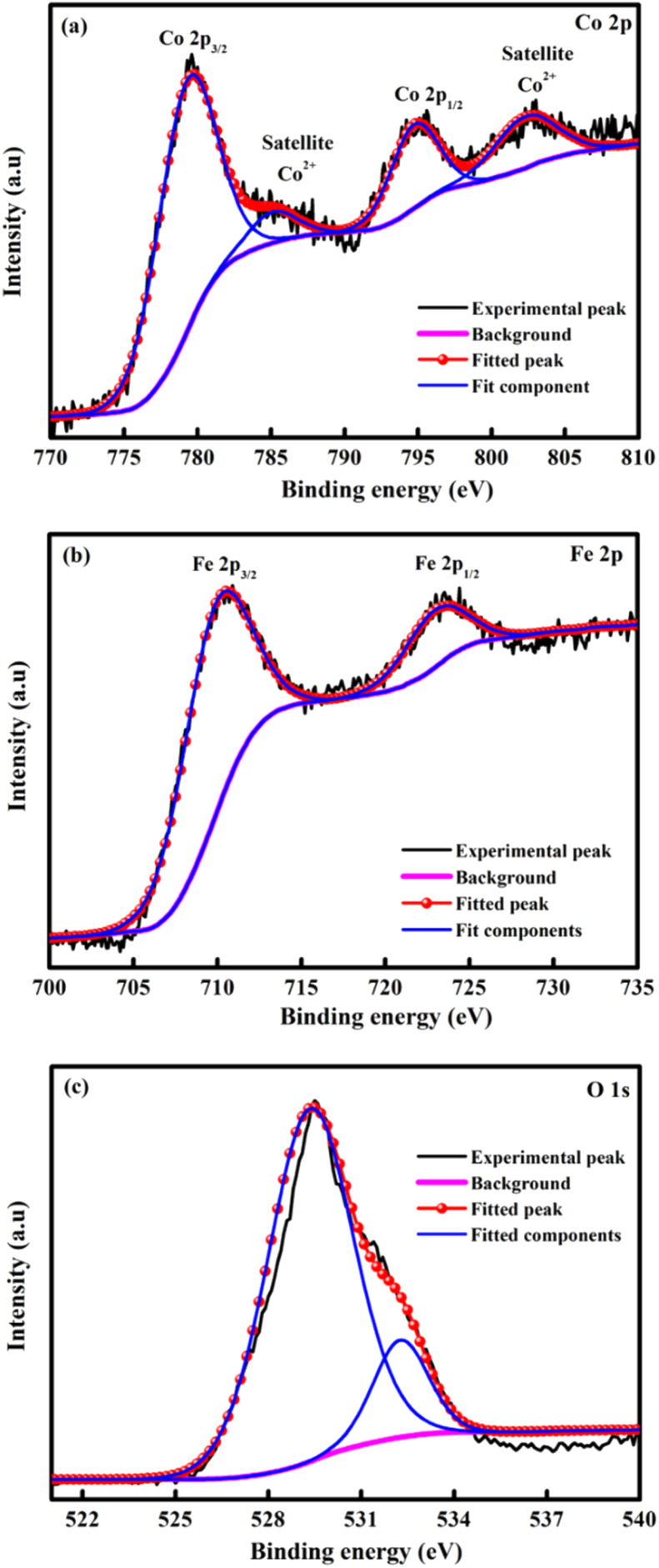
XPS spectra of the survey scan of (a) Co 2p, (b) Fe 2p and (c) O 1s of the CoFe_2_O_4_ for M800.

XPS spectroscopy was explained the elemental composition of CoFe_2_O_4_ and displays the existence of the Co, Fe and O element as showed in Fig. 4. The deconvoluted spectra of the Co 2p (Fig. 4a) spectra show peaks due to Co 2p_3/2_ and Co 2p_1/2_ at binding energy of 779.71 and 794.99 eV respectively.^56^ In addition, the satellites peaks at 785.38 eV and 802.83 eV indicated the presence of unpaired 3d electron of the high spin Co^2+^.^57,58^ In Fig. 4b exposed Fe 2p spectrum and displayed the Fe 2p_3/2_ and Fe 2p_1/2_ peaks at 710.54 and 723.58 eV, respectively. These results support the presence of Fe^3+^ in the inverse spinel CoFe_2_O_4_.^58^ The two peaks at 529.42 and 532.31 eV in a single O 1s fine spectra (Fig. 4c) can be considered as the metal–O bond and consistent with oxygen in the defect, respectively.^56,59^

**Fig. 5:**
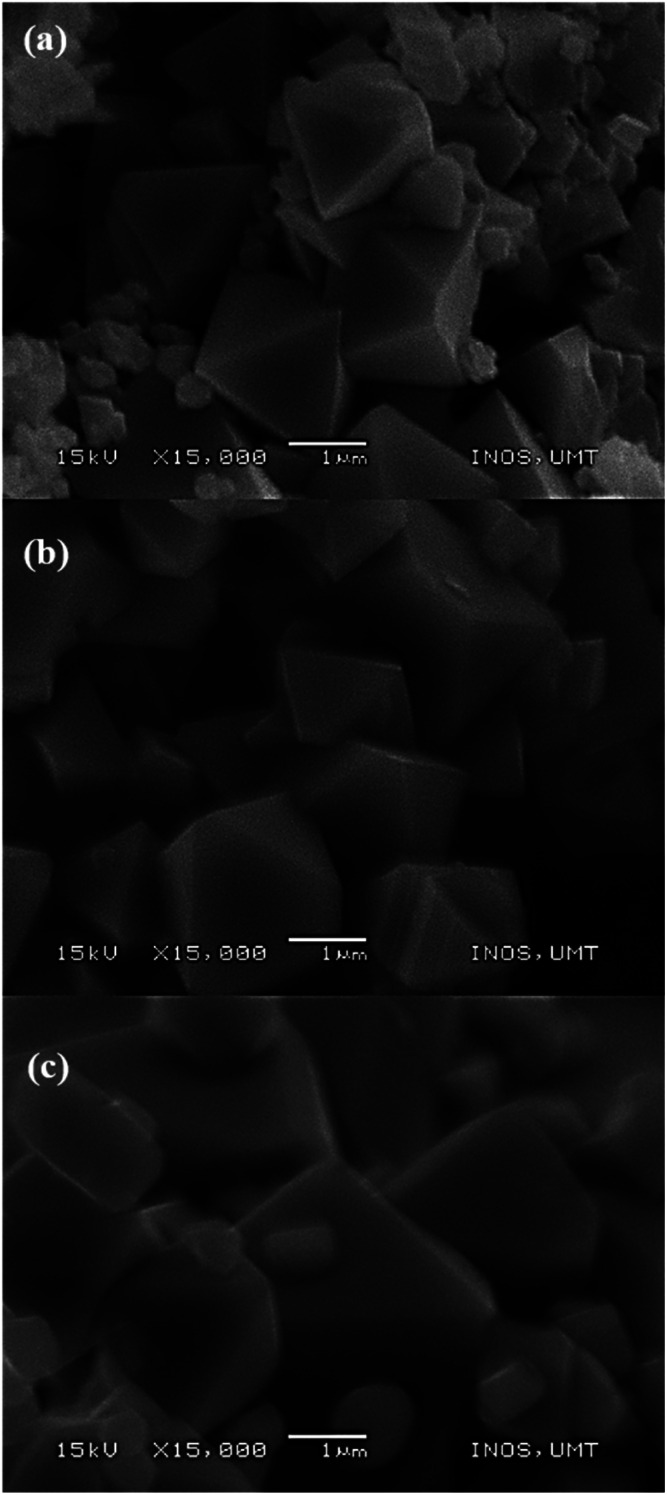
SEM image of CoFe_2_O_4_ for (a) M700, (b) M800, and (c) M900.

SEM images (Fig. 5) showed a remarkable morphological change as the calcination temperatures increased with average particle size ranging from 1.1 to 3.6 μm. Sample M700 (Fig. 5a) shows an octahedron shape with a particle size of ∼1.1 μm, and sample M800 (Fig. 5b) shows a well-defined octahedral shape, with a faceted surface and size of about ∼2.27 μm. As the temperature increased to 900 °C (M900 (Fig. 5c)), the particle sizes increased to ∼3.64 μm and the morphology became flattened, giving rise to new facets. This condition appears inevitable, primarily because of the interaction between magnetic particles at higher calcination temperatures.^60,61^

**Fig. 6:**
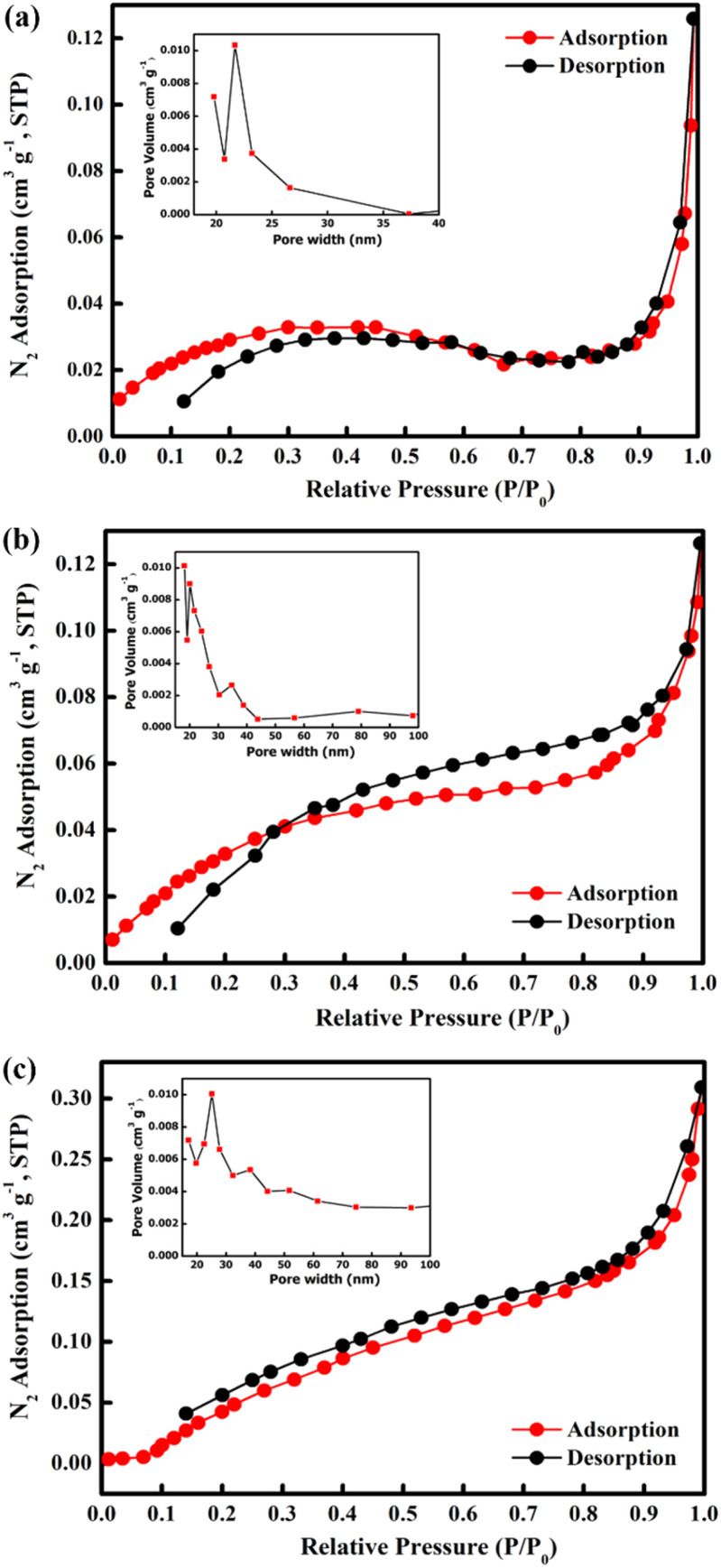
N_2_ adsorption–desorption isotherms and BJH pore size distribution curves (inset) of CoFe_2_O_4_ for (a) M700, (b) M800 and (c) M900.

The Brunauer–Emmett–Teller (BET) surface area of the samples was determined using nitrogen adsorption–desorption isotherms measured at 77.3 K (Fig. 6). From the obtained isotherms, all the samples show type IV adsorption isotherms which indicate mesoporous structures. Furthermore, all samples show H3 hysteresis loop which show the characteristic of slit shape features.^62,63^ The open loop at the isotherm may be caused by slow adsorption at narrow pores which exhibited from slit shape features.^63^ The specific surface areas of M700, M800, and M900 were found to be 2.6017, 3.6244, and 7.7535 m^2^ g^−1^, respectively. In addition, the measured pore volumes of the samples were 0.0020 cm^3^ g^−1^ for M700, 0.0032 cm^3^ g^−1^ for M800 and 0.0082 cm^3^ g^−1^. It is clear that higher calcination temperatures resulted in an increase in the BET surface area due to structural and morphological changes, indicating the emergence of a new facet as shown in the SEM image.^64,65^

**Fig. 7:**
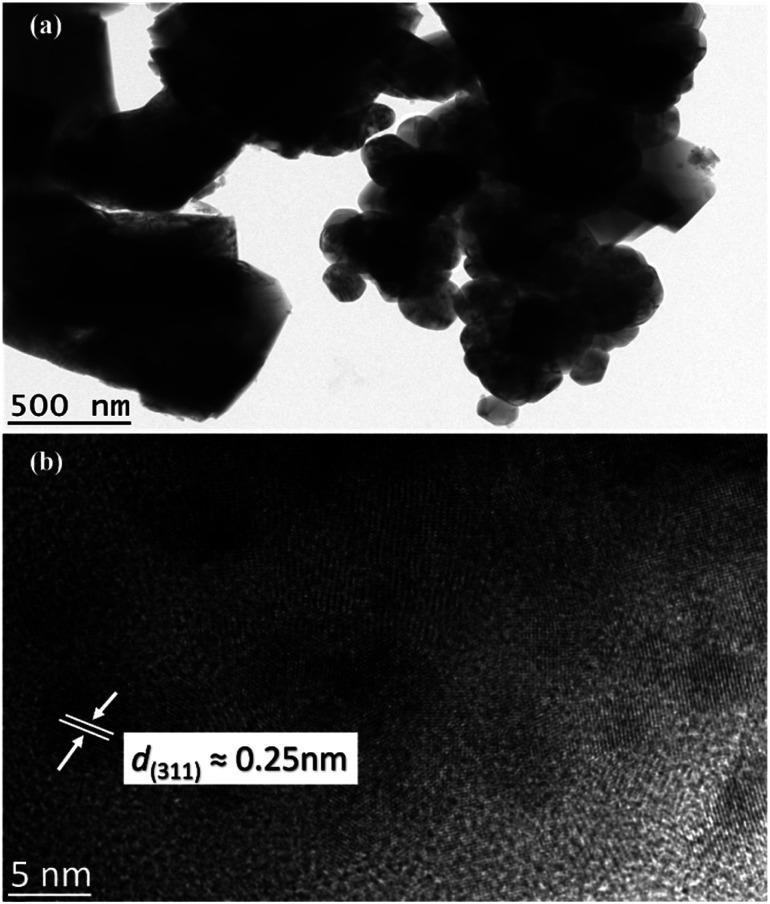
TEM images of M900 at (a) low magnification and (b) high-resolution TEM image of the CoFe_2_O_4_.

Further analysis was conducted using TEM images (Fig. 7). The crystalline CoFe_2_O_4_ structure demonstrates that the sample M900 possessed dense agglomerates, as illustrated in Fig. 7a. Lattice fringes of CoFe_2_O_4_ (Fig. 7b) indicate an interplanar spacing of 0.25 nm belonging to the (311) plane with a cubic phase, which agreed well with the XRD data.

**Fig. 8:**
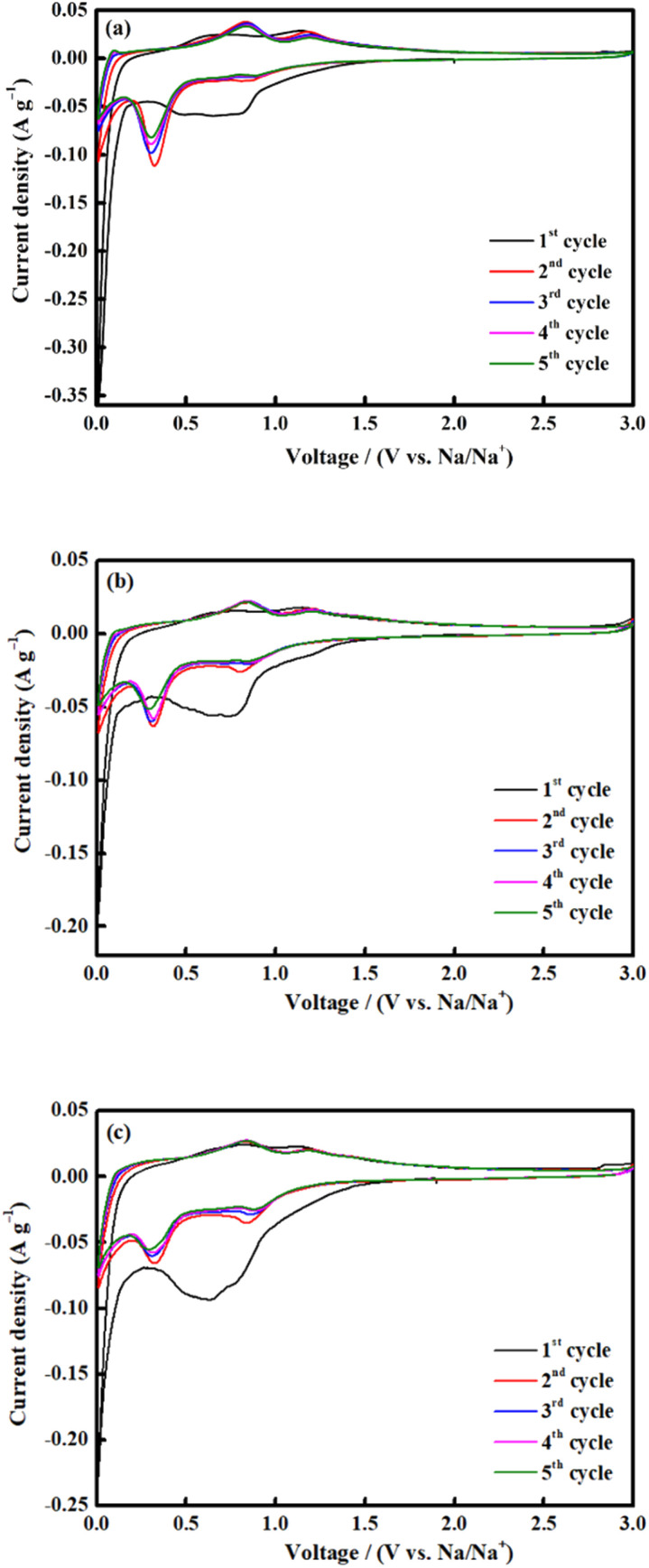
CV measurements of CoFe_2_O_4_ (a) M700, (b) M800 and (c) M900 at a scan rate of 0.1 mV s^−1^ (*vs.* Na/Na^+^).

CV was conducted for all electrodes between 0.01 and 3.0 V at a scan rate of 0.1 mV s^−1^ (Fig. 8). Throughout the first scan, all electrodes showed a broad-ranging cathodic peak at 0.6 V, consistent with the irreversible emergence of a solid electrolyte interface (SEI); electrolyte deterioration causes a significant irreversible loss of capacity during the first discharge process.^33,66^ The shift between 0.3 and 0.8 V during the subsequent cycle is attributed to the reduction of Fe^3+^ and Co^2+^ to Fe^0^ and Co^0^, respectively, and the reversible reaction to form Na_2_O (eqn (2)):^67,68^

2 CoFe_2_O_4_ + 8Na^+^ + 8e^−^ ↔ Co + 2Fe + 4Na_2_O

**Fig. 9:**
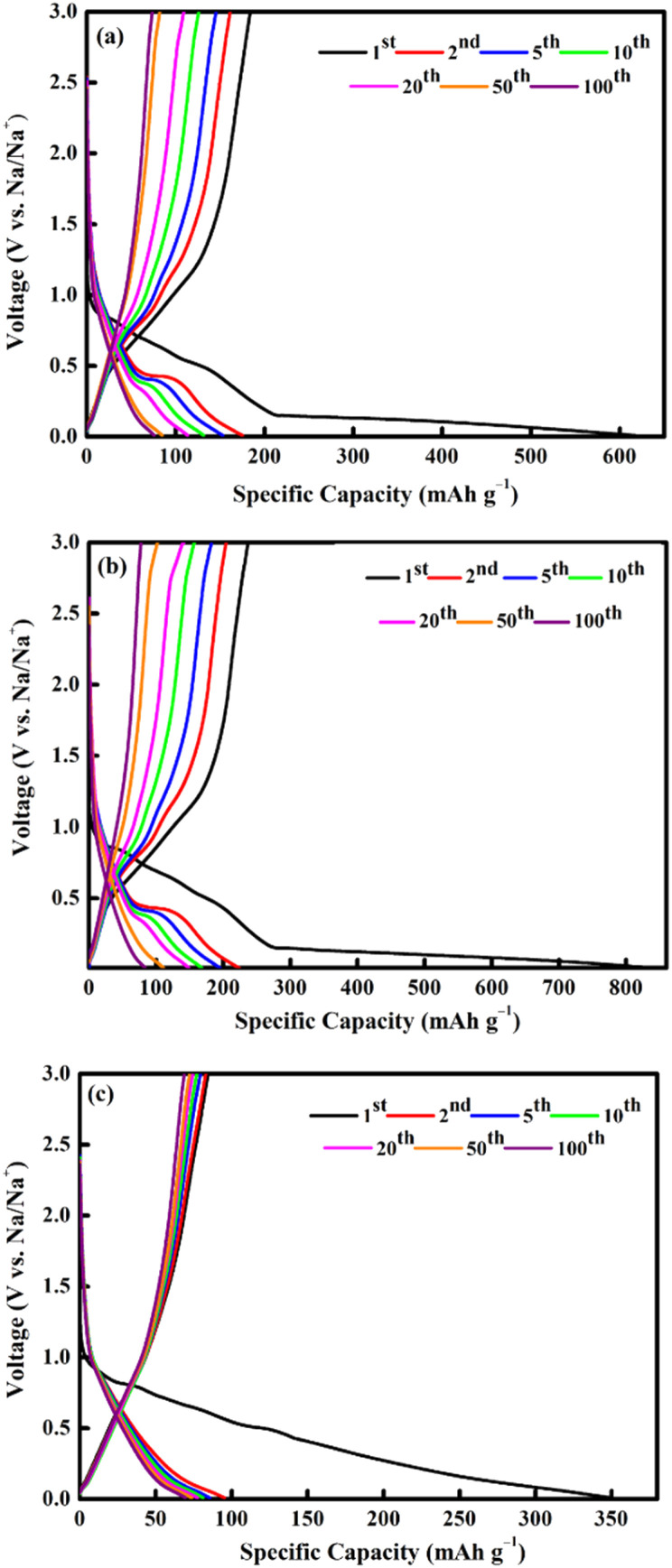
Galvanostatic charge/discharge profiles of CoFe_2_O_4_ (a) M700, (b) M800, and (c) M900 at 0.1 A g^−1^.

In the anodic process, the oxidation peaks at 0.8 and 1.2 V are attributed to the reformation of CoFe_2_O_4_*via* the oxidation of Fe^0^ and Co^0^ to Fe^3+^ and Co^2+^, respectively.^69,70^ All the CV curves almost overlapped during the subsequent cycle, indicating high reversibility of the electrochemical reaction.^33,71^ The selected cycles of the charge and discharge profiles for all electrodes at a current density of 0.1 A g^−1^ is shown in Fig. 9. The charge and discharge plateau for all electrodes is aligned with the CV peaks. The initial capacity of discharge and charge capacities of the electrode are 617 mA h g^−1^ (M700), 839 mA h g^−1^ (M800), and 350 mA h g^−1^ (M900), respectively. Based on these result, M800 electrode deliver higher discharge capacity due to the uniform morphology, suggesting that large contact interface between electrolyte and electrodes, which lead to high irreversible Na^+^ consumption.^72^ Contrarily, the large particle size required a longer time for ion transfer into the particles and faces diffusion limitation of Na^+^ within a single large particle.^65,73^ All electrodes display irreversible capacity loss owing to the formation of the SEI layer and electrolyte degradation during the first cycle.^74,75^ However, there is difference for the second discharge curves of the sample M900 and sample M800 and M700 due to considerable loss in specific capacity of the M900 sample.^76^ This result agreed well with the capacity value of M900 sample which is lower that M800 and M700.

**Fig. 10:**
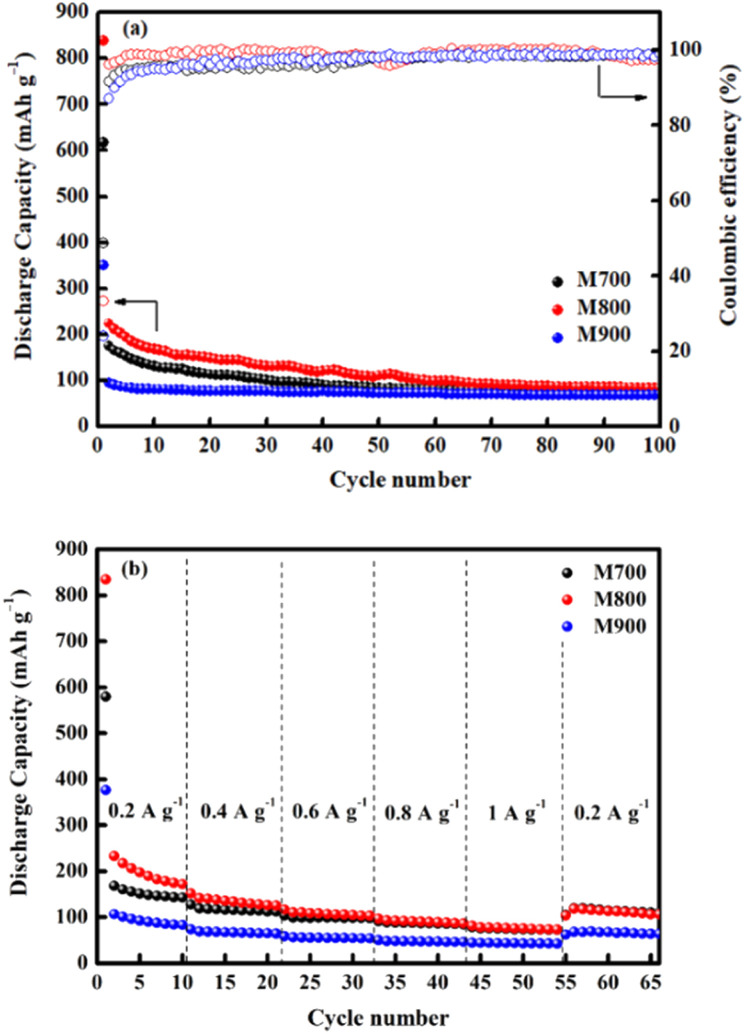
Electrochemical performances of the CoFe_2_O_4_ (M700), CoFe_2_O_4_ (M800), and CoFe_2_O_4_ (M900) electrodes; (a) cycling performance and corresponding coulombic efficiency up to 100 cycles at 0.1 A g^−1^ and (b) rate capability at current densities of 0.2, 0.4, 0.6, 0.8, and 1.0 A g^−1^.

Fig. 10a demonstrates the cycling behaviour of all electrodes at a current density of 0.1 A g^−1^. At the initial cycle, the M800 electrode exhibits the highest discharge capacity (839 mA h g^−1^), followed by the M700 (617 mA h g^−1^) and M900 (350 mA h g^−1^) electrodes. For the M800 electrode, a preserved discharge capacity of 98 mA h g^−1^ was calculated after 100 cycles, whereas the discharge capacity for the M700 (76 mA h g^−1^) and M900 (69 mA h g^−1^) electrodes. The reversible discharge capacity of the M800 electrode was 224 mA h g^−1^ after the second cycle and continued to decline throughout the 100 cycles, which was possibly due the activation and stabilisation processes within the electrode.^15^ In this regard, the observed capacity values of the M800 electrode remain high compared to the other electrodes. Similar trends were also observed for the M700 and M900 electrodes. After 100 cycles, the discharge capacities of the M700 and M900 electrodes were 76 mA h g^−1^ and 69 mA h g^−1^, respectively. According to previous reported,^77–79^ fast capacity fading of materials due to structure collapse and dissolution of materials may occur in electrolyte decomposition. As a result, the improvement in cycling stability of materials is attributed to a delay in structure decay. The specific capacity retained by the M800 electrode after 100 cycles was 88%, compared to 87% and 80% retained by the M700 and M900 electrodes, respectively. Clearly, the initial coulombic efficiencies were 48%, 33% and 24% for the M700, M800 and M900 electrodes, respectively, owing to uncontrolled SEI layer formations. After several cycles, all the electrodes demonstrated high coulombic efficiencies of more than 99% as the SEI layer formation stabilised during cycling.^80^ The rate capability of all the electrodes was also determined at different current rates, ranging from 0.2 to 1.0 A g^−1^ (Fig. 10b). The M800 electrode delivered the discharge capacities of 171, 125, 103, 87, 73 and 108 mA h g^−1^ at the current densities of 0.2, 0.4, 0.6, 0.8, 1.0 and 0.2 A g^−1^, respectively. Even though the rate returned to 0.2 A g^−1^, the discharge capacity of M800 electrode could still display the maximum reversible capacity, suggesting stable cycling performance. However, the consecutive cycling performances of the M700 and M900 electrodes were unsatisfactory. After 66 cycles at various charge and discharge rates, the discharge capacity of the M800 electrode at 0.2 A g^−1^ remained 108 mA h g^−1^, representing approximately 87% capacity recovery. Hence, the improved cycle and rate performance of the M800 electrode is superior to that of the M700 and M900 electrodes, may be due to the well-defined octahedral shape of M800 delivers sufficient active sites for Na-ion, thus reducing the electron and ion transport pathways.

The improved electrochemical performance of CoFe_2_O_4_ (M800) could be assigned to the high crystallinity and homogeneous distribution of particles, leading to a high surface area,^81^ facilitating electrode–electrolyte interaction and affording increased active sites for electrochemical reactions.^82,83^ The well-dispersed particles are beneficial for excellent performance because they provide a short transport length and a substantial contact area between the active material and electrolyte.^84,85^ In this regard, the sample must afford a high surface area for promoting the adsorption and storage of Na-ions.^86^ M900 presented a large particle size as seen from the SEM image, which in turn severely affect the performance because it is difficult for ions to diffuse into bulk materials. It suggests that increasing the length of diffusion pathways of Na-ions and resulting in unsatisfactory CoFe_2_O_4_ behaviour. This method is well recognised for its cost-effective preparation because the products can be produced in large quantities in a short session.^87^ Scientifically, the salt melts during the preparation method owing to the high rate of ion absorption and high ability to dissolve, which can speed up the rate of the reactions.^88^ To the best of our knowledge, systematic investigations on the parameters influencing the formation and characteristics of CoFe_2_O_4_ during the molten salt method are still lacking. Overall, the discharge capacity of CoFe_2_O_4_ discovered in this study is preferable to those previously reported (Table 2). The synthesis of molten salts at low temperatures represents a template and surfactant free, cost-effective, simple and an efficient method for large-scale production. As a result of this study, new insights can be gained for future studies on CoFe_2_O_4_ as an anode material for Na-ion batteries.

**Table 2:** Comparative electrochemical performances of CoFe_2_O_4_ anode for Na-ion batteries synthesized from various techniques

Sample	Synthesis method	Current density (A g^−1^)	Discharge capacity (mA h g^−1^)/cycle	Reference
CoFe_2_O_4_	Hydrothermal	0.1	300/1^st^ cycle	23
CoFe_2_O_4_	Hydrothermal	0.05	700/1^st^ cycle	72
CoFe_2_O_4_	Hydrothermal	0.05	500/1^st^ cycle	34
CoFe_2_O_4_	Annealing metal–organic framework	0.05	573/1^st^ cycle	33
CoFe_2_O_4_	Molten salt	0.1	839/1^st^ cycle	This work

## 4. Conclusions

CoFe_2_O_4_ was successfully synthesised using the molten salt method, followed by calcination at 700 °C, 800 °C and 900 °C. The synthesis approach used provides a straightforward and practical way for industrial production. The powder phases, structures, chemical composition and morphology are characterised through XRD, Raman spectroscopy, FTIR, XPS, SEM, BET and TEM. The electrochemical results indicate that the M800 electrode showed excellent performance as an anode material for Na-ion batteries, which could be attributed to the homogeneity, uniform octahedral morphology, and high crystallinity of the material. The M800 electrode revealed a high initial discharge capacity (839 mA h g^−1^ at 0.1 A g^−1^) and retained the capacity (98 mA h g^−1^) after 100 cycles. The capacitive retention was ∼88% after 100 cycles, demonstrating a good rate capability and cycling stability during the insertion and de-insertion of Na-ions. These findings indicate that this strategy may provide an innovative approach to improving the electrochemical behaviour of CoFe_2_O_4_ electrodes for use in Na-ion batteries.

## Author contributions

The manuscript original draft was written by S. U. M. S. U. M., and N. H. I. investigated the molten salt synthesis of disordered spinel CoFe_2_O_4_ with improved electrochemical performance for sodium-ion batteries and developed conceptualisation. S. U. M., N. H. I., H. M. Y., F. M. D., S. R. M., and L. N. developed the methodologies. The project was supervised by N. H. I. The manuscript has been reviewed and edited by all contributing authors.

## Conflicts of interest

The authors declare that they have no known competing financial interest or personal relationships that could have appeared to influence the work reported in this paper.

## Supplementary Material
